# Structure-function analysis of the ATPase domain of African swine fever virus topoisomerase

**DOI:** 10.1128/mbio.03086-23

**Published:** 2024-02-27

**Authors:** Wenhua Kuang, Yan Zhao, Jinyue Li, Zengqin Deng

**Affiliations:** 1 Key Laboratory of Special Pathogens and Biosafety, Wuhan Institute of Virology, Center for Antiviral Research, Chinese Academy of Sciences, Wuhan, Hubei, China; 2 University of Chinese Academy of Sciences, Beijing, China; 3 Hubei Jiangxia Laboratory, Wuhan, Hubei, China; Case Western Reserve University School of Medicine, Cleveland, USA

**Keywords:** African swine fever virus, topoisomerase, ATPase domain, crystal structure

## Abstract

Type II topoisomerase utilizes the energy from ATP hydrolysis to alter DNA topology during genome replication and transcription. The ATPase domain of this enzyme is required for ATP hydrolysis and plays a crucial role in coupling DNA binding and ATP turnover with the DNA strand passage reaction. The African swine fever virus (ASFV) specifically encodes a topoisomerase II (topo II), which is critical for viral replication and an attractive target for antiviral development. Here, we present a high-resolution crystal structure of the ASFV topo II ATPase domain complexed with the substrate analog AMPPNP. Structural comparison reveals that the ASFV topo II ATPase domain shares a conserved overall structure with its homologs from eukaryotes and prokaryotes but also has three characteristic regions, including the intra-molecular interface formed by the ATP-lid and QTK loop as well as helix α9, the K-loop in the transducer domain, and the antennae-like α-helix at the ATP binding domain. Mutating the key residues within these three regions impairs or abolishes the basal and DNA-stimulated ATPase activities and reduces or eliminates the relaxation activity of the holoenzyme. Our data indicate that all three regions are functionally important for the ATPase and relaxation activities and strongly suggest that ATP hydrolysis, DNA binding, and strand passage are highly coupled and managed by the allosteric coordination of multiple domains of the type II topoisomerase. Moreover, we find a promising druggable pocket in the dimeric interface of the ASFV topo II ATPase domain, which will benefit future anti-ASFV drug development.

**IMPORTANCE:**

The ATPase domain of type II topoisomerase provides energy by hydrolyzing ATP and coordinates with the DNA-binding/cleavage domain to drive and control DNA transport. The precise molecular mechanisms of how these domains respond to DNA binding and ATP hydrolysis signals and communicate with each other remain elusive. We determine the first high-resolution crystal structure of the ATPase domain of African swine fever virus (ASFV) topo II in complex with AMPPNP and biochemically investigate its function in ATPase and DNA relaxation activities. Importantly, we find that mutations at three characteristic regions of the ASFV ATPase domain produce parallel effects on the basal/DNA-stimulated ATPase and relaxation activities, implying the tight coupling of the ATP hydrolysis and strand passage process. Therefore, our data provide important implications for understanding the strand passage mechanism of the type II topoisomerase and the structural basis for developing ATPase domain-targeting antivirals against ASFV.

## INTRODUCTION

Type II topoisomerases are ATP-dependent enzymes that can relax supercoils and resolve knots and catenates by generating double-strand breaks and rejoining the DNA fragment (1, 2). They exist universally in organisms from three-domain life and some viruses with double-stranded DNA genomes, such as bacteriophages and nucleocytoplasmic large DNA viruses (NCLDVs) (3). By regulating the topological states of DNA in cells, type II topoisomerases play essential roles in fundamental cellular processes, including DNA replication, transcription, recombination, and proper chromosome segregation. According to their structures and catalytic mechanisms, type II topoisomerases are divided into type IIA and IIB. As the primary type II topoisomerase, type IIA includes three groups of enzymes, named topo II, DNA gyrase, and topo IV, respectively (4). Among them, topo II contains a single polypeptide that forms a homodimer. In contrast, DNA gyrase and topo IV possess two separate subunits (B and A), which together assemble into a B_2_A_2_ heterotetramer. Despite different sequences and organizations, all type IIA holoenzymes share conserved functional domains, including an N-terminal ATPase domain and a central DNA-binding/cleavage domain, followed by a C-terminal coiled-coil (5–8). These domains cooperate to complete the DNA relaxation reaction and maintain the topological homeostasis of DNA in living organisms.

Type II topoisomerases comprise three interfaces that separately constitute its three corresponding molecular gates: the N-gate formed by the ATPase domain, the DNA-gate formed by the DNA-binding/cleavage domain, and the C-gate formed by the C-terminal coiled-coil. It is believed that type II topoisomerases utilize a common strand passage mechanism to accomplish the catalytic reaction and involve the hydrolysis of two ATP molecules. At the initial stage of the reaction cycle, the DNA-binding/cleavage domain binds and bends a gate segment (G-segment), then two ATPase domains bind two ATP molecules and trap a transport segment (T-segment), accompanied by the dimerization of these two ATPase domains. Next, the G-segment is cleaved by the catalytic tyrosine of the DNA-binding/cleavage domain, and the T-segment passes through the open DNA-gate; both steps are triggered by the hydrolysis of the first ATP. At the late stage, the C-gate opens for the release of the T-segment, followed by religation of the G-segment and closure of the DNA-gate with the hydrolysis of the second ATP. At the end of the reaction, the enzyme returns to its original state due to the release of the ADP molecules (9, 10).

The ATPase domain provides the energy for the reaction by hydrolyzing ATP, thus indispensable for the strand passage activity of type II topoisomerase. Structural and functional information of the isolated ATPase domain is primarily from several eukaryotic and prokaryotic type II topoisomerases (11–18). The available structures of the ATPase domain from different type II topoisomerases show a similar overall architecture consisting of an N-terminal ATP-binding domain, also known as the GHKL (Gyrase, Hsp90, Histidine Kinase, MutL) domain, and a C-terminal transducer domain. The GHKL domain can bind an ATP molecule and a metal ion Mg^2+^ in the active site, which is responsible for ATP hydrolysis. Previous studies indicated that the GHKL domain can dimerize after ATP binding and lead to the closure of the N-gate (16, 18). The transducer domain is thought to be able to transduce structural signals induced by the ATP binding and hydrolysis to the downstream DNA-binding/cleavage domain, thus guiding the DNA strand passage process. In addition, the transducer domain can also couple DNA binding to the ATPase activity and strand passage by interacting with the G-segment (5). However, the precise molecular mechanism of how the enzyme responds to the DNA binding and ATP hydrolysis signals through inter-domain cooperation remains elusive. Type II topoisomerases are successful targets for developing antibacterial and anticancer drugs owing to their essential role in cell survival (19–23). One class of agents targeting the ATPase domain are the bisdioxopiperazines, including ICRF-187 and its derivatives, which can hinder the ATP turnover by locking the dimer interface of the ATPase region and consequently inhibiting the relaxation activity of the holoenzyme (11, 16).

African swine fever virus (ASFV) is a highly pathogenic agent that causes serious hemorrhagic diseases and death in both domestic pigs and wild boars, with mortality rates approaching 100%. The African swine fever disease caused by ASFV infection had spread worldwide and resulted in enormous economic losses to the global pig industry (24–26). However, to date, there are no effective vaccines or specific therapeutic drugs against ASFV. ASFV is a large double-stranded DNA virus with an icosahedral structure and belongs to the NCLDV family. It encodes its own topoisomerase, topo II, which plays a critical role in viral transcription and replication (27–29). In our previous study, we determined two cryo-EM structures of ASFV topo II and revealed unique structural features important for its function (30), which provides a foundation for the understanding of virus topoisomerase. However, the cryo-EM structures did not resolve the ATPase domain, probably owing to the flexible connection between the ATPase domain and the following DNA-binding/cleavage domain, which precludes the structure determination of the ATPase domain. Here, we present the high-resolution crystal structure of the isolated ATPase domain from ASFV topo II in complex with AMPPNP (a non-hydrolyzable ATP analog). Combining structure-based mutagenesis and biochemical analysis, we identify several structural regions that are important for the basal and DNA-stimulated ATPase activity, and all the regions are closely linked to the relaxation activity in the context of the holoenzyme. Our data provide structural and functional insights into the strand passage mechanism of type II topoisomerase and offer a basis for developing antivirals targeting the ATPase domain of ASFV topo II.

## RESULTS

### Overall structure of the ASFV topo II ATPase domain in complex with AMPPNP

**Fig 1:**
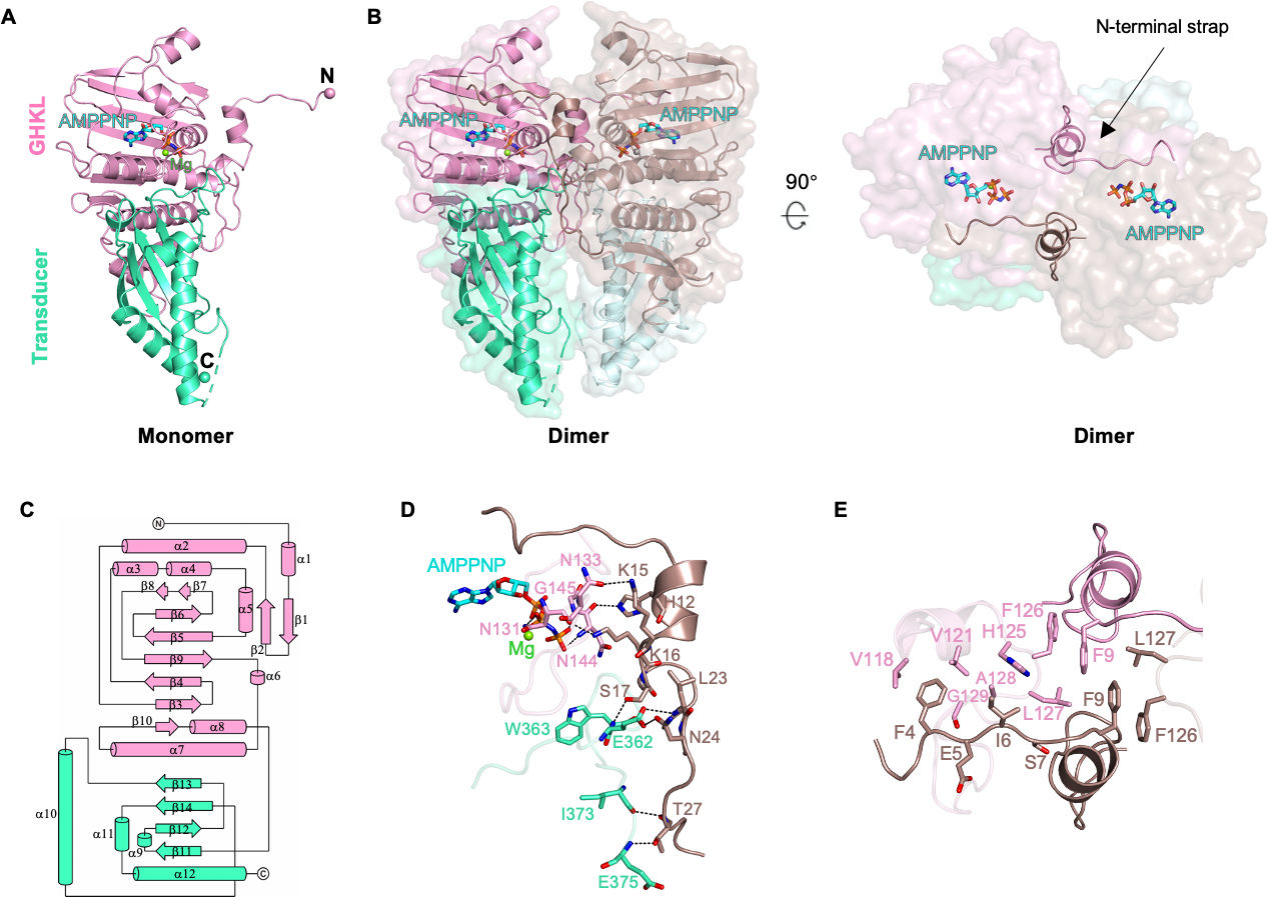
Overall structure of the ASFV topo II ATPase domain complexed with AMPPNP. (**A**) and (**B**) Cartoon representations of the ASFV topo II ATPase domain monomer and dimer. The GHKL and transducer domain are colored pink or brown and green or light cyan, respectively. The semi-transparent surface representation is also shown for the dimer. The AMPPNP molecule is shown as sticks, and the bound Mg^2+^ in the active site is shown as a green or gray sphere. The N-terminal strap from each protomer that locks the dimer interface is highlighted and labeled. (**C**) Topological diagram of ASFV topo II ATPase domain. (**D**) and (**E**) Dimer interface interactions mediated by the N-terminal strap. Hydrogen bonds are labeled with dotted black lines.

To structurally characterize the ASFV topoisomerase ATPase domain, we crystallized the ATPase domain (residues 1–414) in the presence of AMPPNP. The crystal structure of the ASFV topoisomerase ATPase domain was determined at 2.20 Å resolution in the space group *P*3_1_, with four ATPase molecules in the crystallography asymmetric unit (Table S1) displaying highly consistent global conformations [root-mean-square deviation (RMSD) value of 0.1 Å for all Cα]. Similar to other reported type II topoisomerase ATPase structures, ASFV ATPase contains an N-terminal GHKL domain and a C-terminal transducer domain (Fig. 1A), together assembling into a heart-shaped dimeric complex (Fig. 1B). The GHKL domain (residues 1–263) comprises a characteristic nucleotide-binding fold that consists of eight antiparallel β-strands backed against eight α-helices with different lengths and harbors several conserved residues responsible for metal-ion and ATP binding and ATP hydrolysis. The transducer domain (residues 264–414) contains four antiparallel β-strands packed against four α-helices (Fig. 1A and C), which is believed to be able to transduce the ATP hydrolysis signals to the downstream DNA-binding/cleavage domain.

The ASFV ATPase dimer contains a primary interface mainly formed by the side-edge of the GHKL domain and locked by a long N-terminal strap from each monomer (Fig. 1B), which buries a roughly 2,000 Å^2^ of molecular surface per subunit. The N-terminal strap from one monomer makes extensive hydrogen bonds and hydrophobic interactions with both the GHKL and transducer domain from the other monomer (Fig. 1D and E), which contributes greatly to the stability of the dimeric conformation. Besides the interface formed by the GHKL domain, the transducer domain constitutes a relatively smaller interface with ~500 Å^2^ of molecular surface per subunit. Previous studies demonstrated that the two interfaces collectively constitute the N-gate essential for T-segment transport.

### ASFV topo II ATPase domain shares a conserved architecture with other type IIA topoisomerase ATPase domains

**Fig 2:**
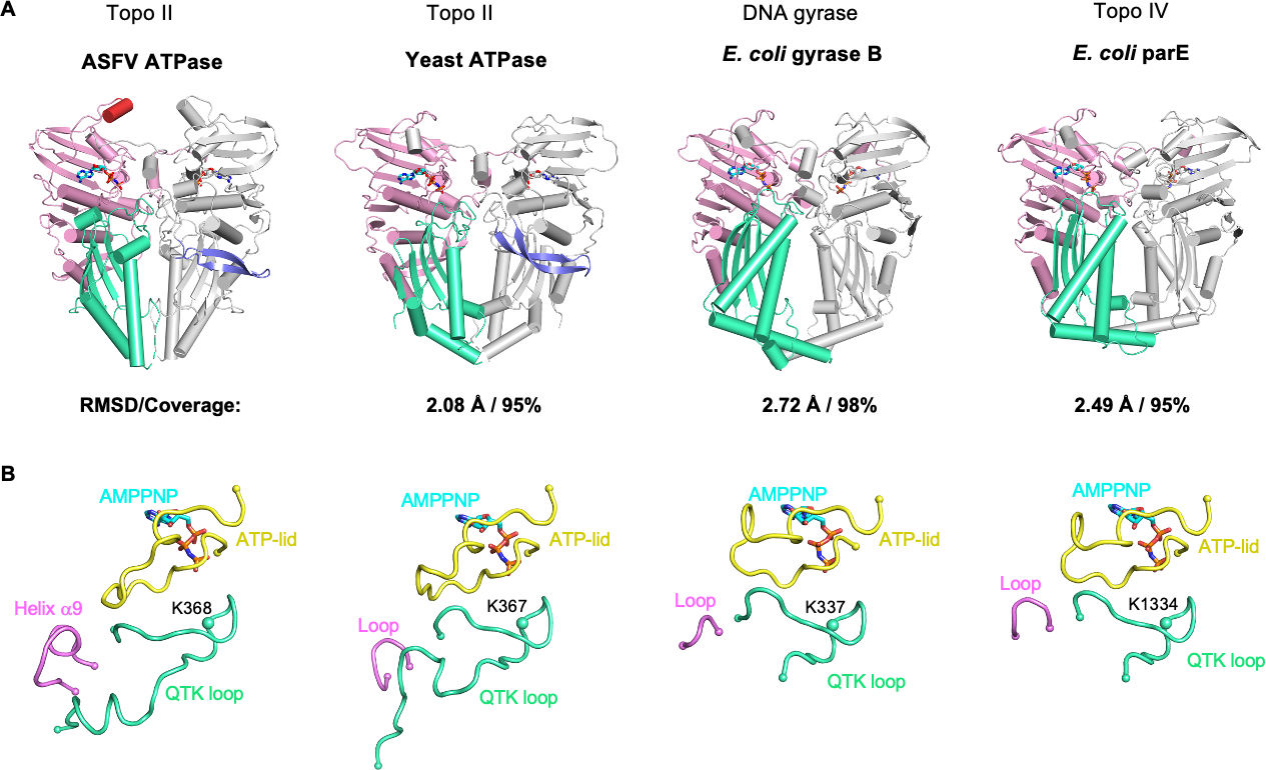
ASFV topo II ATPase domain shares a conserved overall fold with those from eukaryotic and prokaryotic topo II. (**A**) Comparison of the overall structures of ASFV topo II and other type II topoisomerase ATPase domains. The ATPase domain dimer is represented in the cartoon. One protomer is colored as in Fig. 1A, and the other is shaded gray. The β-hairpin uniquely existed in ASFV topo II and eukaryotic type II topoisomerases is highlighted in one protomer and colored in slate. The RMSD and residue coverage values are indicated using the dimer of the ASFV topo II domain as a reference. PDB entries: 1PVG (yeast topo II ATPase domain); 1EI1 (*E. coli* gyrase B); and 1S16 (*E. coli* parE). (**B**) Structural comparison of the intra-molecular interface formed by the ATP-lid, QTK loop, and helix α9 in different type II topoisomerases. The ATP-lid of ASFV and yeast topo II ATPase domains adopt a semi-open conformation, while those of *E. coli* gyrase and parE show a relatively closed conformation. The AMPPNP is shown as the reference for the active site. The *α*-carbon atom of the conserved switch lysine on the QTK loop is labeled and shown as a sphere.

Structural comparison shows that the ASFV ATPase domain exhibits a similar overall architecture with other type II topoisomerase ATPase domains from eukaryotes and prokaryotes (RMSD 2.08–2.72 Å, 95%–98% residue coverage), with the highest similarity to the yeast topo II ATPase domain (Fig. 2A). In addition, the ASFV ATPase domain also contains a β-hairpin that uniquely exists in eukaryotic enzymes, indicating that the viral type II topoisomerase is more closely related to those of eukaryotes than prokaryotes. Despite the conservation of overall structure, some diverse regions are noted in the structures of different ATPase domains, in particular, the interface formed by the ATP-lid from GHKL domain, the QTK loop, and helix α9 from the transducer domain (Fig. 2B). The ATP-lid contributes to AMPPNP binding by several conserved residues and serves as a lid that covers over the AMPPNP. The ATP-lid adopts a relatively closed conformation in *Escherichia coli* gyrase B and parE, whereas a semi-open conformation in ASFV and yeast ATPase domains. The QTK loops from different ATPase domains are similar in conformation but distinct in length. Notably, they bear an absolutely conserved lysine that binds to the AMPPNP and is thought to be a molecular switch response to ATP hydrolysis. The helix α9 is adjacent to the ATP-lid and QTK loop and unique in the ASFV topo II ATPase domain, which is replaced with a short loop in those of other ATPase domains (Fig. 2B). Overall, the ATP-lid and QTK loop bear conserved residues required for AMPPNP binding, indicating their important roles in ATPase activity. Moreover, all three structural elements constitute the domain interface, implying their specific roles in regulating the conformational change upon enzyme reaction. Besides the differences in the above-mentioned domain interface, the ASFV domain also contains an additional α-helix like a “tentacle” at the apex of the dimer (Fig. 2A), which is absent in other homologs and presumably associated with functionality.

### Binding site of AMPPNP in the ASFV topo II ATPase domain

**Fig 3:**
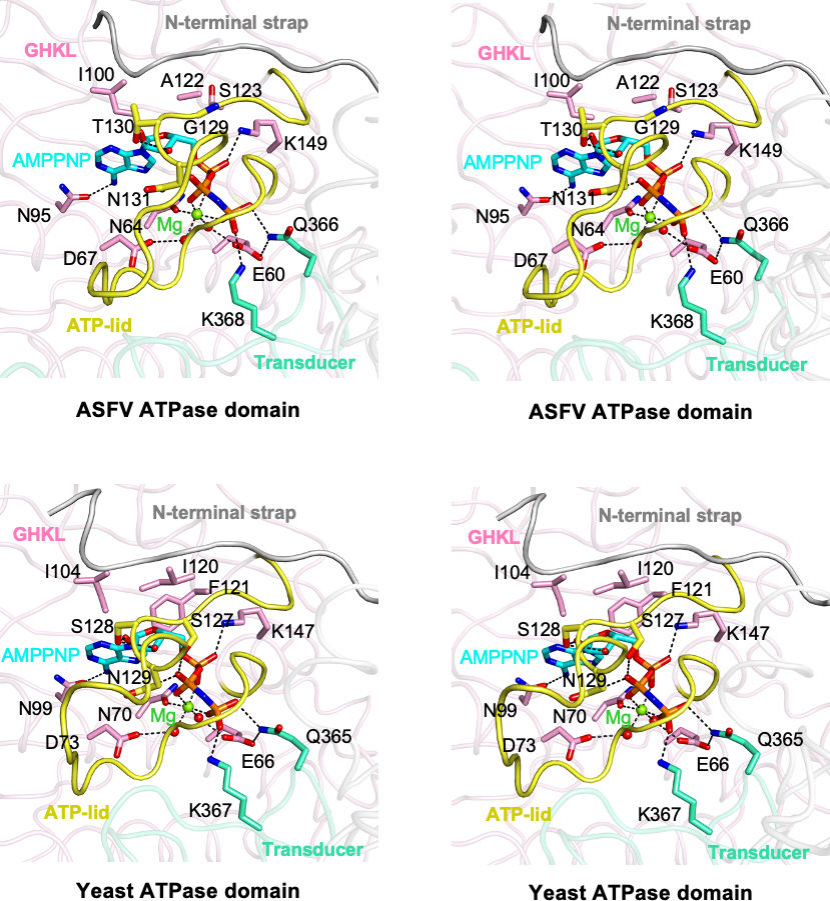
Comparison of the binding site of the AMPPNP in ASFV and yeast topo II ATPase domains in stereo-pair images. Protein structures are shown using ribbon style, and the domains are colored as in Fig. 1A. The AMPPNP bound in the active site is shown as sticks. Magnesium ions and water molecules are shown as green and red spheres, respectively. Key residues involved in AMPPNP binding are labeled and shown as sticks. The ATP-lid and the N-terminal strap are labeled and colored in yellow and gray, respectively.

In the structure of ASFV topo II ATPase domain bound to AMPPNP, the nucleotide binds in the active site and engages in an intact pocket mainly formed by the ATP-lid, the N-terminal strap, strands β4−β5 and β9, and helices α2 and α4–α5 of the GHKL domain. Besides, the QTK loop from the transducer domain also contributes to the pocket by making a hydron bond (Q366) and a critical salt bridge (K368) with the γ-phosphate of AMPPNP (Fig. 3, upper panels). As seen in the previous study, a single Mg^2+^ ion was observed in the active site, which is octahedrally coordinated by each oxygen of the three phosphates of AMPPNP, the side chain of N64, and two water molecules bridged by D67 and E60. The α- and β-phosphate groups also make additional contacts with the side chains of K149 and N131 on the ATP-lid, respectively. The ribose ring is stabilized by direct hydrogen bonds between the hydroxyl groups and the side chain of T130 on the ATP-lid. The adenine moiety is held in place through hydrogen bonding to N95, together with van der Waals contact with I100. Among the residues involved in AMPPNP binding, E60 is supposed to act as a catalytic base that activates the water molecule to allow nucleophilic attack of the γ-phosphate, while K368 stabilizes the transition state of ATP hydrolysis reaction.

The binding mode of AMPPNP in the active site of the ASFV ATPase domain is largely consistent with that reported in the yeast topo II ATPase domain (Fig. 3, lower panels), and the involved residues, including E60, D67, N64, Q366, and K368, are highly conserved among type II topoisomerases (Fig. S1), indicating their pivotal roles in Mg^2+^ and AMPPNP binding. Nevertheless, structural differences are also observed in the conformation of the ATP-lid between the two structures. In the ASFV topo II ATPase domain, the apex portion (residues 133–138) of the ATP-lid flips outward and close to the transducer domain, whereas in the yeast topo II ATPase domain, this region (residues 131–136) is close to the active site. Together, these observations suggest general uniformity in the active site configuration and ATP-binding mode of the type II topoisomerase ATPase domain. However, some discrepancies still exist in the conformation of the associated structural elements, which might be related to the function of the individual enzyme.

### Structure-based design of ASFV topo II ATPase domain mutations

**Fig 4:**
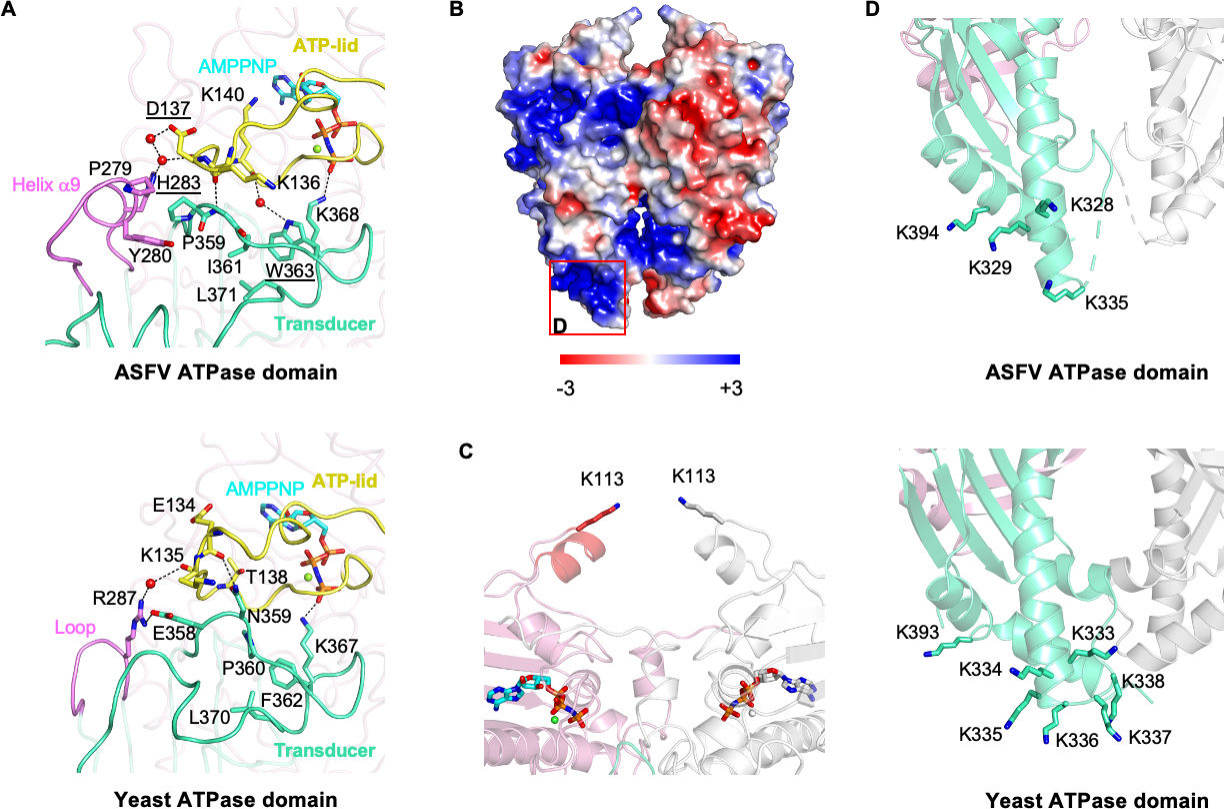
The characteristic structural regions of the ASFV topo II ATPase domain. (**A**) Comparison of the intra-molecular interface of ASFV and yeast topo II ATPase domains. The color scheme for the ATP-lid, QTK loop, and helix α9 is the same as in Fig. 2B. Residues and water molecules involved in the interaction are shown as sticks and spheres, respectively. Residues chosen for mutagenesis are highlighted with an underline. (**B**) Electrostatic surface potential of the ASFV topo II ATPase domain. The region corresponding to the K-loop with a high positive potential (blue) is marked by a red square. Scale bar: −3 kT/e in red to +3 kT/e in blue. (**C**) Close-up view of the unique antennae-like α-helices in the ASFV topo II ATPase domain dimer. The basic residue K113 sitting oppositely at the apex of each monomer is labeled and shown as sticks. (**D**) Comparison of the K-loop of ASFV and yeast topo II ATPase domains (PDB entry: 4GFH). One protomer is in color and the other one in gray. The basic lysine residues potentially involved in DNA binding are labeled and shown as sticks. In the ASFV topo II ATPase domain, the K394 (K393 in yeast topo II) located on the α-helix adjacent to the K-loop is likely to engage in DNA binding.

To understand the molecular action mechanisms of the ASFV ATPase domain, we dissected some special structural regions that might be associated with the ATPase activity and signal transduction, including the intra-molecular interface formed by the ATP-lid, QTK loop as well as helix α9, a positively charged patch at the bottom of the transducer domain, and the unique pair of antennae-like α-helices at the apex of the dimer. The intra-molecular interface between the GHKL and transducer domains is mainly stabilized via direct or water-mediated hydrogen bonds among the ATP-lid, QTK loop, and helix α9. The hydrophobic contacts within the transducer domain, especially the helix α9 and QTK loop, also contribute to the conformational stability of the interface (Fig. 4A). Similar interaction networks are also observed in the domain interface of yeast topo II ATPase domain. However, the interacting residues are variable in the two structures (Fig. 4A; Fig. S1), implying the importance of the intra-molecular interface for the enzyme. The positively charged patch at the bottom of the transducer domain contains several basic residues that are equivalent to the K-loop, except for the K394, required for DNA-stimulated ATP hydrolysis found in the yeast topo II ATPase domain (Fig. 4B and D). Interestingly, the additional α-helices at the apex of the dimer harbor a pair of lysine facing each other (Fig. 4C), just like an antenna applied to receive a molecular signal. To investigate the function roles of these regions in the ATPase activity and the relaxation activity, we designed three sets of mutations, aiming to break the contacts between the intra-molecular interface and the putative interactions with the DNA substrate (Table 1).

**TABLE 1:** Activities of wild-type (WT) ASFV topo II and mutants

Structural regions	Constructs	Relaxation activity (%)^*a*^	Basal ATPase activity of isolated ATPase domain (fold stimulation)^*b*^	Basal ATPase activity of holoenzyme (fold stimulation)^*b*^
	WT	96	0.21 (1.6)	3.77 (2.3)
Active site	Y800A N64A	5 13	N/A0.04 (0.6)	3.20 (1.3) 0.70 (0.9)
Intra-molecular interface	D137A H283A W363A	65 67 13	0.16 (2.0) 0.19 (1.9) 0.08 (1.3)	2.20 (1.7) 3.44 (1.7) 1.06 (1.4)
Antennae-like α-helices	K113A	82	0.15 (1.9)	2.68 (1.8)
K-loop	K328/K329/K335/K394E	4	0.19 (0.8)	2.14 (1.0)

^*a*^ Relaxation activity is presented as the percentage of relaxed pUC19 DNA.

^*b*^ ATPase activity is presented as the turnover rate by measuring the released inorganic Pi. Values are the mean of three independent experiments.

### *In vitro* relaxation activities of the ASFV topo II holoenzyme mutants

**Fig 5:**
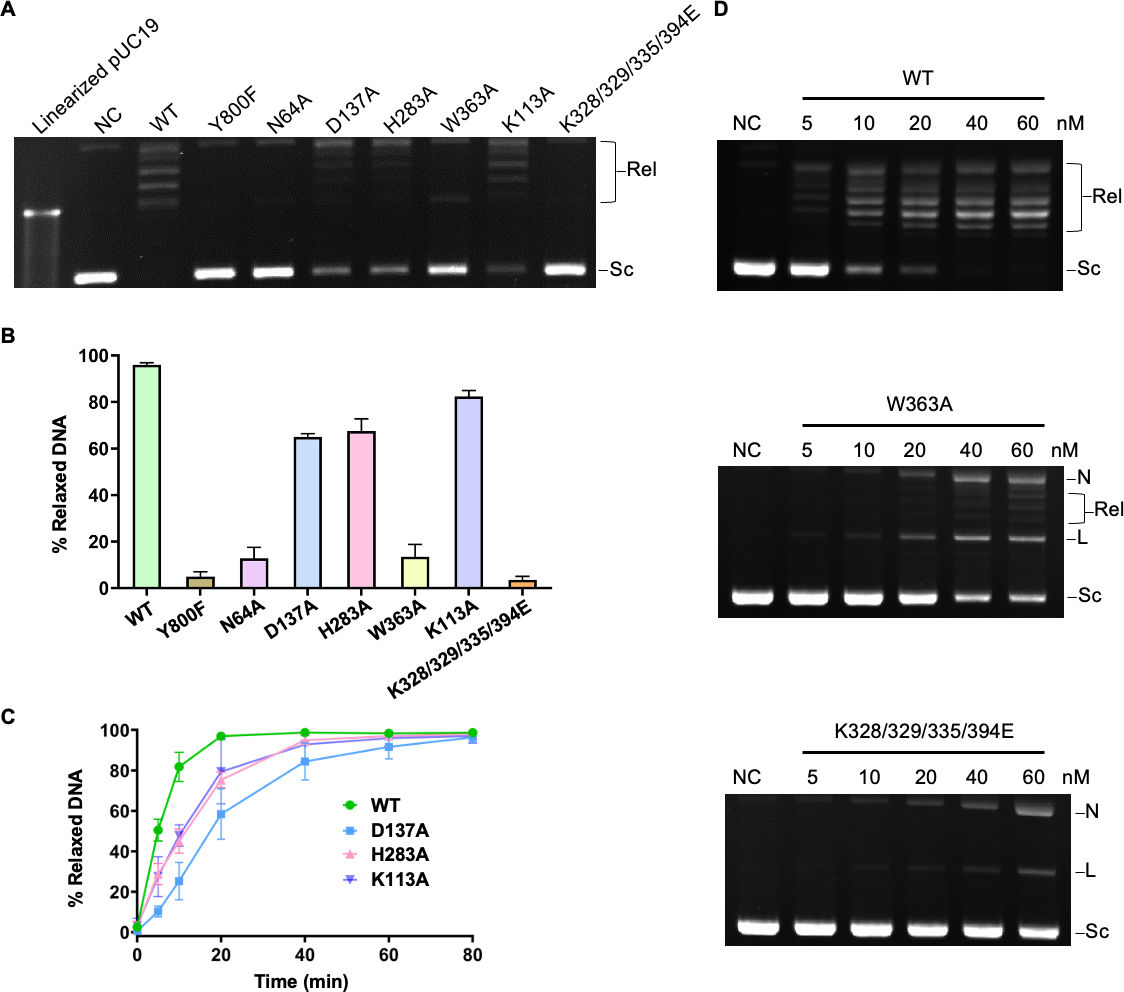
Mutations at the three characteristic regions impair DNA strand passage of ASFV topo II. (**A**) and (**B**) Relaxation activity of ASFV topo II and mutants. The pUC19 plasmids were incubated with 15 nM WT or indicated mutant protein for 40 min at 30°C. Reaction without enzyme was conducted as the negative control (NC). For the K-loop combined mutant, all the four lysine residues were mutated to glutamate. (**C**) Time course of relaxation activity of WT ASFV topo II and selected mutants. The reaction was performed as in Fig. 5A and stopped at the indicated time point. (**D**) Concentration-dependent relaxation activity of WT ASFV topo II and selected mutants. The pUC19 plasmids were incubated with WT or mutant protein (indicated concentration) for 40 min at 30°C. For panels A and D, the gel of one representative experiment is shown. For panels B and C, data are presented as mean values ± SD for three independent experiments. Supercoiled (Sc), relaxed (Rel) topoisomers, nicked (**N**), and linear (**L**) DNA are indicated.

All the designed mutations were introduced into the ASFV topo II holoenzyme to assess their effects on the relaxation activity with negatively supercoiled DNA. The active site residues, including the catalytic tyrosine Y800 of the DNA-binding/cleavage domain and the key Mg^2+^ coordinated residue N64 of the ATPase domain, were chosen as the negative controls. Expectedly, the active site mutants Y800A and N64A abolished the relaxation activity (Fig. 5A and B). The intra-molecular interface mutant W363A greatly reduced the relaxation activity at the normal enzyme concentration (15 nM) and showed moderate activity only at a high enzyme concentration (60 nM) (Fig. 5A and D). The other two intra-molecular interface mutants, D137A and H283A, and mutant K113A for the antennae-like α-helices retained but exhibited an apparently lower activity than the wild type (WT) (Fig. 5A through C). Interestingly, the combined mutations at the K-loop and K394 abolished the activity, albeit at a high enzyme concentration (Fig. 5A and D). Notably, two distinct bands appeared with increased concentrations of W363A and K-loop mutants (Fig. 5D, middle and bottom panels), which, respectively, correspond to the position of nicked and linear DNA, suggesting mutations at W363 and the K-loop did not affect the cleavage activity but impaired the relaxation activity. These data indicate that the intra-molecular interface residue W363 and the K-loop are critical for the DNA strand passage activities of ASFV topo II, while the intra-molecular interaction (D137-H283) and the antennae-like α-helices also have impacts on the activity.

### Mutations in the three characteristic regions affect the ATPase activity of ASFV topo II

**Fig 6:**
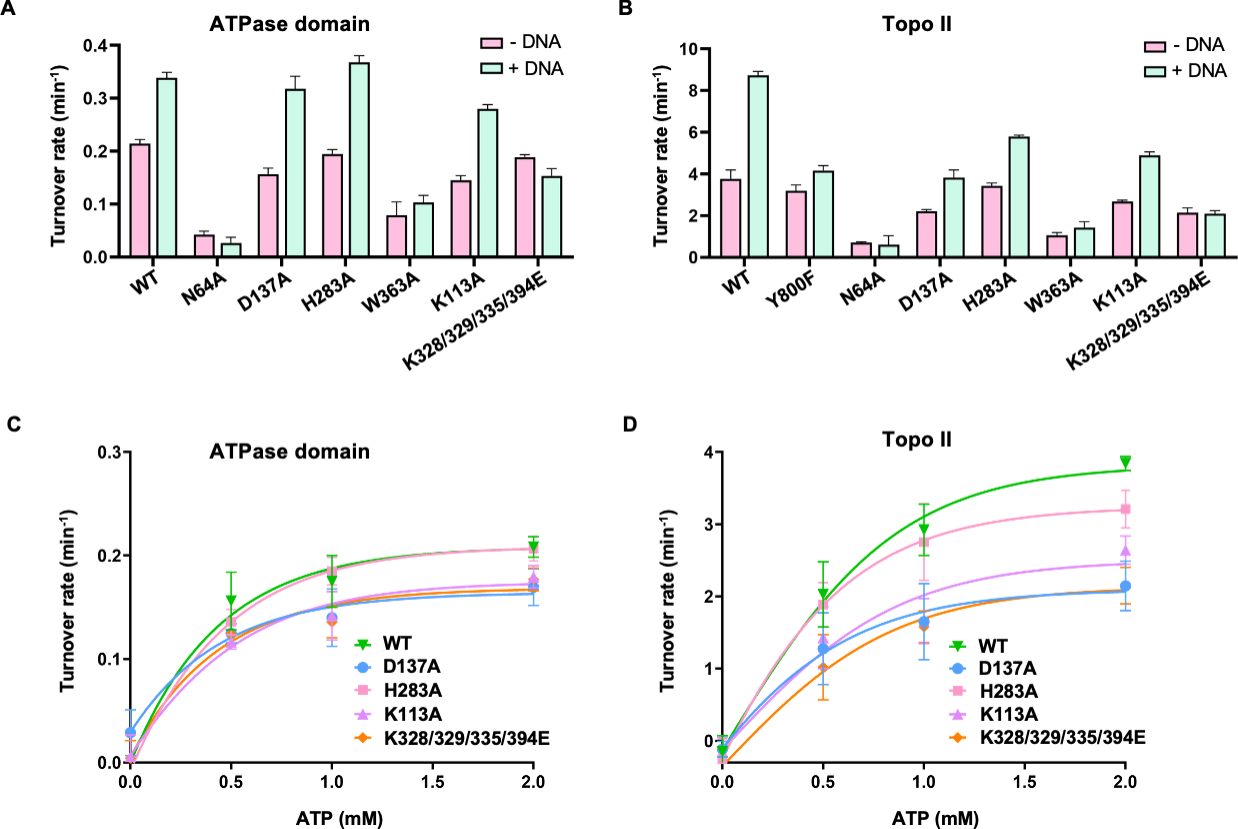
Mutations at the three characteristic regions affect the ATPase activity of ASFV topo II. (**A**) and (**B**) Basal and DNA-simulated ATPase activities of the isolated ATPase domain and the holoenzyme. Reaction mixture containing enzyme (5 or 0.3 µM) and 2 mM ATP was incubated at 37°C for 30 min in the presence or absence of 40 ng/µL of pUC19 plasmids. (**C**) and (**D**) Concentration-dependent basal ATPase activity of the isolated ATPase domain and the holoenzyme. Basal ATPase activity of the WT and mutants was determined at an increasing concentration of ATP (0, 0.5, 1.0, and 2.0 mM). Data are presented as mean values ± SD for three independent experiments.

Since the DNA strand passage process of the holoenzyme is highly dependent on the ATP hydrolysis activity, we further examined the basal and DNA-stimulated ATPase activities of the isolated ATPase domain and the holoenzyme mutants. For the isolated ATPase domain, the WT protein displayed a basal ATPase activity that is stimulated 1.6-fold upon the addition of pUC19 plasmids (Fig. 6A). However, mutations at the active site residue N64 and the intra-molecular interface residue W363 resulted in significantly reduced basal and DNA-stimulated ATPase activity. Mutations at D137, H283, and K113 had no or less effects on both the basal and DNA-stimulated ATPase activities, although the two mutants D137A and K113A exhibited an apparently lower activity than the WT. Unlike the other mutants, the K-loop combined mutant had a comparable basal ATPase activity to WT, whereas the addition of DNA gave no stimulation effect, indicating the K-loop is essential for DNA-stimulated ATP hydrolysis (Fig. 6A). Under the context of the holoenzyme, the activity profiles for all mutants in the presence or absence of the DNA are largely consistent with those of the isolated ATPase domain, except for the H283A mutant, which exhibited lower DNA-stimulated ATPase activity compared with WT (Fig. 6B). Notably, the WT holoenzyme showed a higher DNA-stimulated ATPase activity than the isolated ATPase domain. In contrast, mutations in the context of the holoenzyme resulted in greater inhibitory effects on the DNA-stimulation activity than that of the isolated ATPase domain (Fig. 6A and B; Table 1). To confirm the effects of the mutations on the intrinsic ATPase activity in detail, we performed the experiment at a range of ATP concentrations, and all mutants except H283A still exhibited reduced basal ATPase activity (Fig. 6C and D). Together, mutations at the three characteristic regions both reduced or abrogated the basal and DNA-stimulated ATPase activity and, in turn, affected the relaxation activity, indicating they play important roles in coupling the ATP hydrolysis to strand passage.

## DISCUSSION

The ATPase domain can both trap the T-segment and provide the energy necessary to drive strand passage, which plays an essential role in the function of type II topoisomerase. Here, we report the high-resolution crystal structure of the ATPase domain of ASFV topo II, which reveals a very similar overall fold to those of the eukaryotic and prokaryotic type II topoisomerase. It shares the highest structural similarity with that of yeast topo II and displays almost identical active site configuration (Fig. 2A and 3 ). Particularly, it harbors a special β-hairpin loop that is found only in eukaryotic enzymes. These observations are largely consistent with the scenario of the viral origin of eukaryotic type II topoisomerases supported in previous studies (31, 32). Despite the similar overall architecture, structural divergences are still observed in the organization of domain interface formed by the ATP-lid, QTK loop, and helix α9 in different type II topoisomerases (Fig. 2B). Since the three structural elements bridge the ATP binding and transducer domains together, they probably play important roles in the signal transduction of the ATP hydrolysis via allosteric communication. In addition, the ATPase domain of type II topoisomerases forms a cavity with different sizes in the center of the dimer that presumably accommodates the T-segment. We observed that the cavity in the ASFV ATPase domain is smaller than those in other homologs (Fig. S2), suggesting that the enzyme might be more sensitive to T-segment and undergo more profound conformational changes during strand passage. Overall, different type IIA topoisomerases likely share a common ATP hydrolysis mechanism due to the conserved overall fold of the ATPase domain, and the variation in the structures may contribute to the catalytic properties of the individual enzyme.

We identified three characteristic regions in the ASFV topo II ATPase domain based on structural analysis. We further conducted mutagenesis analysis to investigate their functions in ATP hydrolysis and strand passage (Fig. 4). Our data show that mutating the domain interface residues has different impacts on the basal/DNA-stimulated ATPase activity and the relaxation activity (Fig. 5 and 6). Among the mutated residues, W363 on the QTK loop forms hydrophobic stacking with the switch residue K368 and links with the ATP-lid by water-mediated hydrogen (Fig. 4A). Mutating W363 might interrupt the conformational stability of the ATP-lid and the QTK loop and, in turn, directly affect the binding of K368 with the γ-phosphate of the nucleotide, which explains why this mutation has the most impact on the activity. Mutations disrupting the D137-H283 interaction also partially reduced the ATPase activity and the relaxation activity (Fig. 5 and 6). Previous studies showed that the transducer domain suffers significant conformational changes during the ATP hydrolysis, including the whole domain module rotates outward by ~10° relative to the center of the dimer and the QTK loop retracts back and shifts away from the ATP-binding site for the release of the product Pi (12, 16). Residues D137 and H283 are located at the joint of the domain interface and form a flexible hydrogen bond mediated by water molecules (Fig. 4A), suggesting it likely engages the transducer domain movement through structural rearrangement. Of note, mutation at D137 showed more inhibitory effect than H283 on the activity, probably because D137 contributes to preserving the conformational stability of the ATP-lid. Our results indicate that domain interface interactions contribute to both the structural stability and mobility of the ASFV topo II ATPase domain and play an important role in the ATP hydrolysis cycle.

The DNA-stimulated effect in ATPase activity has been shown in several type II topoisomerases (5, 14, 33, 34). In our study, the ATPase activity of both the isolated ATPase domain and the holoenzyme can be stimulated by pUC19 plasmids (Fig. 6A and B), consistent with the results reported in previous studies. The domain interface mutants for the isolated domain have lower DNA-stimulated ATPase activity compared to that of WT, which may be explained by its low basal activities (Fig. 6A and C), while the decrease in the activity in the holoenzyme may also relate to the impairment in the function of the signal transduction (Fig. 6B and D). As an exception, the ATPase activity of the K-loop combined mutant failed to be stimulated upon the addition of DNA and exhibited no apparent relaxation activity (Fig. 6A and B), suggesting the K-loop is directly relevant to the DNA-stimulated activity. The results largely agree with that reported in the yeast topo II study (5), in which the K-loop can couple the ATPase hydrolysis with strand passage by directly contacting the G-segment. Since K394 is relatively conserved among the viral and eukaryotic type II topoisomerases (Fig. S1), it is likely involved in DNA binding. Previous studies revealed that both the G and T segment binding can stimulate the ATPase activity (5, 14, 35). Thus, the activity of the isolated ATPase domain and the holoenzyme could theoretically be stimulated by the DNA with different modes. However, our results show that mutation of the K-loop completely abrogates the DNA-stimulated activity for both the isolated ATPase domain and the holoenzyme (Fig. 6A and B), indicating pUC19 plasmids serve as a G-segment involved in the stimulation process in the two cases. Mutation at K113 on the additional antennae-like α-helices impaired the basal and DNA-stimulated ATPase activities (Fig. 6A and B), indicating that this region is also associated with ATP hydrolysis. Since the two K113 residues oppositely sit on top of the N-gate (Fig. 4C), it might be involved in capturing the T-segment by sensing or responding to DNA binding. Interestingly, we found that the WT holoenzyme displayed higher DNA-stimulated activity compared to the isolated ATPase domain (Fig. 6A and B; Table 1), suggesting the coupling effects of the enzyme via DNA binding and multiple domains’ cooperation, which may explain why mutations in the holoenzyme instead resulted in greater inhibitory effects. Overall, our results indicate that mutations on the three characteristic regions in the ASFV topo II ATPase domain have parallel effects on the basal/DNA-stimulated ATPase activity and relaxation activity (Fig. 5 and 6 ), strongly suggesting the process of ATP hydrolysis, DNA binding, and strand passage are closely coupled and managed by allosteric regulation of the individual domain of the holoenzyme.

Type II topoisomerases have been recognized as an ideal target for anticancer and antibacterial drugs. Several drugs targeting the specific catalytic steps of type II topoisomerases have been developed; for instance, the bisdioxopiperazine inhibitors (ICRF-187 and ICRF-193) can halt the catalytic cycle by trapping the ATPase domain in a closed conformation. Previous studies showed that the ICRF-187 could inhibit the relaxation activity of yeast and human topo II with half-maximal inhibitory concentration (IC_50_) values at the micromolar level (36–38). Considering that the ATPase domain is structurally conserved among the type II topoisomerases, we conducted an *in vitro* relaxation activity assay to assess whether ICRF-187 inhibits ASFV topo II. Unexpectedly, the results showed that ICRF-187 has no inhibitory activity on the ASFV topo II, even at a high concentration (millimolar level) (Fig. S3B). To explore the possible reasons, we superimposed the ASFV topo II ATPase domain with the crystal structure of the yeast topo II ATPase-ICRF-187 complex. It showed that the ICRF-187 binding site is relatively conserved between yeast and ASFV ATPase domain. However, a variable residue M18 in the ASFV topo II ATPase domain produces an obvious steric hindrance with the piperazinedione ring moiety of ICRF-187 (Fig. S3C). This M18 residue is replaced with a threonine with a short side chain in the yeast and other eukaryotic topo II, which is associated with the ICRF-187 by van der Waals interaction. Besides, the variable residue W19 may also hinder the entry of the ICRF-187 because its large side chain narrows the entrance of the dimer interface pocket (Fig. S3C). We constructed three mutants, including two single mutants (M18T and W19Y) as well as one double mutant (M18T/W19Y), and performed the relaxation activity inhibition assays. Unexpectedly, the double mutant lost the relaxation activity, precluding further relaxation activity inhibition analysis (Fig. S3A). We performed inhibition assays using the two single mutants, which preserve comparable relaxation activity as WT. The results show that ICRF-187 still has no inhibitory effect on the relaxation activity of the two single mutants (Fig. S3B). All these observations suggest that the current bisdioxopiperazine inhibitors might not apply to ASFV topo II. Nevertheless, the anti-ASFV drug screen and design targeting this pocket are promising. Molecular compound binding to this pocket can act as a molecular glue to lock the dimeric conformation of the ASFV topo II ATPase domain, thus preventing the ATP hydrolysis cycle. Besides ICRF-187, a recent work showed arctiin and genistein, small-molecule metabolites of *Bacillus subtilis,* could inhibit ASFV infection, probably by competing for ATP binding to the ATP-binding domain of ASFV type II topoisomerase (39). We performed molecular docking analysis using the crystal structure of the ASFV topo II ATPase domain. The result shows that arctiin exactly binds to the ATP-binding site in a manner similar to that of AMPPNP and makes extensive interactions with the active site residues (E60, H68, N95, P98, I100, T130, N131, and G142) (Fig. S4). These observations suggest that the ATP-binding site is an efficient target for drug development and supports arctiin as an anti-ASFV drug candidate for further exploration.

In summary, we determine the first atomic structure of the ATPase domain of the viral topo II and identify three characteristic regions that have functional relevance for ATP hydrolysis and strand passage. The intra-molecular interface may confer structural stability and flexibility, which adapts the enzyme to accomplish the ATP hydrolysis and signal transduction to the downstream modules. While the K-loop can couple the DNA binding to ATP turnover and further propel the strand passage process. The antennae-like α-helices might act as a molecular sensor and be involved in the T-segment capture. Moreover, our results suggest that type II topoisomerase could couple multiple catalytic steps by domain communications and allosteric regulation. Overall, our work provides important insights into the catalytic mechanism of type II topoisomerases and offers guidelines for developing antivirals targeting the ATPase domain of ASFV topo II.

## MATERIALS AND METHODS

### Protein expression and purification

The DNA fragments encoding the full-length protein (residues 1–1192) and the ATPase domain (residues 1–414) of ASFV topo II (P1192R, Gene ID: 59227094) were cloned into a yeast expression vector pPICZ-B fused with an N-terminal MBP-His10 tag. Single point or combined mutations were introduced into the full-length protein and the ATPase domain of ASFV topo II using site-directed mutagenesis. Protein overexpression was carried out in yeast *Pichia pastoris* (SMD1163H) grown in a minimal medium supplemented with glycerol for a carbon source and methanol for induction (24–48 h at 30°C). The induced cells were harvested and disrupted with a high-pressure cell crusher (Union-Biotech). The ASFV topo II protein purification procedure is similar to that described previously (30). Briefly, the protein was purified by Ni^2+^-chelating affinity chromatography followed by size exclusion chromatography using a Superose 6 Increase 10/300 Gl column. The ATPase domain was prepared using a similar purification strategy as the full-length of ASFV topo II, except using different lysis buffer [20 mM Tris-HCl, pH 8.0, 1 M NaCl, 50 mM imidazole, 10% (vol/vol) glycerol, 0.1% Triton X-100, 1 mM PMSF, and 20 U/mL DNase I), and gel-filtration buffer (20 mM Tris pH 8.0 and 150 mM NaCl) for Superdex 200 10/300 Gl size-exclusion chromatography column. The purified proteins were concentrated with an Amicon concentration device, flash-frozen in liquid nitrogen, and stored as aliquots at −80°C for further use.

### Crystallization, diffraction data collection, and structure determination

Crystallization trials of the ATPase domain were performed by sitting drop vapor diffusion at 16°C. Typically, 8 and 10 mg/mL protein supplemented with 0.5 mM AMPPNP and 5 mM MgCl_2_ were mixed with the precipitant/reservoir solution at a 1:1 vol ratio in 0.6 µL drop volume. Initial crystals appeared in the precipitant/reservoir solution (0.2 M ammonium sulfate, 0.1 M HEPES pH 7.5, and 25% PEG 3350) within 1 week. Large sword-shaped crystals were obtained by optimization of the precipitant concentration (20%–30%, wt/vol) and using the hanging-drop vapor diffusion method. Crystals were harvested, transferred to the crystallization buffer plus 15% (vol/vol) glycerol, and flash-frozen in liquid nitrogen. The X-ray diffraction data were collected at the BL02U1 beamline of Shanghai Synchrotron Radiation Facility with a wavelength of 0.979 Å and temperature of 100 K. At least 180 degrees of data were collected with 0.4° oscillation and exposure of 0.1 s at 100% transmission.

The diffraction data were indexed, integrated, and scaled using the HKL2000 software package (40), followed by reduction analysis with the CCP4 suite (41). The initial model was obtained by the molecular replacement program PHASER (42) using a predicted structure by AlphaFold2 (43) as the search model. Structure refinement was performed iteratively with automated refinement in Phenix (44) and manual model building in Coot (45). The final model contains residues 1–335 and 341–403 with *R*_work_ and *R*_free_ values of 0.176 and 0.220, respectively. The resolved crystal structure is similar to the predicted structure by AlphaFold2 with an RMSD value of 0.72 Å for all Cα (chain B of crystal structure as a reference). The data collection and refinement statistics for the final model are listed in Table S1.

### Relaxation activity assay

For relaxation activity assays, 15 nM or indicated concentration of purified protein was incubated with 25 ng/µL of supercoiled pUC19 plasmids in 160 µL reaction buffer [50 mM Tris-HCl, pH 8.0, 2 mM ATP, 30 mM NaCl, 5 mM DTT, 5 mM MgCl_2_, and 5% (vol/vol) glycerol]. The reaction was conducted at 30°C for 40 min or the indicated time and stopped by adding 1% (wt/vol) SDS and then digested with proteinase K for 30 min at 37°C. The samples were separated by electrophoresis in 1% (wt/vol) agarose gels with TBE buffer, stained with 0.5 mg/mL GoldView, and visualized with UV light. DNA quantification analysis was performed using ImageJ. For the relaxation activity inhibition assay, the ICRF-187 (indicated concentration) was preincubated with the enzyme for 20 min on ice, and then the ATP substrate was added to start the reaction.

### ATPase activity assay

ATPase activity was measured as previously described (46). Briefly, the purified ATPase domain (5 µM) or the full-length ASFV topo II protein (0.3 µM) were incubated with 2 mM or indicated concentration of ATP substrate at 37°C for 30 min in 50 µL assay buffer [20 mM Tris-HCl pH 8.0, 30 mM NaCl, 2 mM ATP, 5 mM DTT, 5 mM MgCl_2_, and 5% (vol/vol) glycerol], in the presence or absence of 40 ng/µL of pUC19 plasmids. The reaction was stopped by adding 100 µL of freshly prepared Solution II (2.86% ascorbic acid, 1 M HCl, 0.48% [NH4]_2_MoO_4_, and 2.86% SDS) and incubated for 10 min on ice. Then, the mixture was added with 150 µL of Solution III (3.5% bismuth citrate, 1 M HCl, and 3.5% sodium citrate) and incubated at 37°C for 10 min. The absorbance values at 710 nm were measured by UV spectroscopy (SpectraMax ABS Plus Microplate Readers, Molecular Devices). Reaction without enzyme was performed as a negative control. The ATP hydrolysis rate was calculated by dividing the released inorganic phosphate by the enzyme concentration and the reaction time using a standard curve established by phosphate standards.

## Data Availability

Atomic coordinate and structure factor for the reported crystal structure have been deposited in the Protein Data bank under accession number 8WWO.

## SUPPLEMENTAL MATERIAL

Supplemental Material mbio.03086-23-s0001.docx Fig. S1-S4 and Table S1.

ASM does not own the copyrights to Supplemental Material that may be linked to, or accessed through, an article. The authors have granted ASM a non-exclusive, world-wide license to publish the Supplemental Material files. Please contact the corresponding author directly for reuse.
