# Supplementary material for: Structure-function analysis of the ATPase domain of African swine fever virus topoisomerase
Source: mBio. 2024 Feb 27;15(4):e03086-23. doi: 10.1128/mbio.03086-23 (PMC11005426; doi:10.1128/mbio.03086-23)
Supplement: Supplemental Material — Fig. S1-S4 and Table S1. [file mbio.03086-23-s0001.docx]

**Supplemental materials**


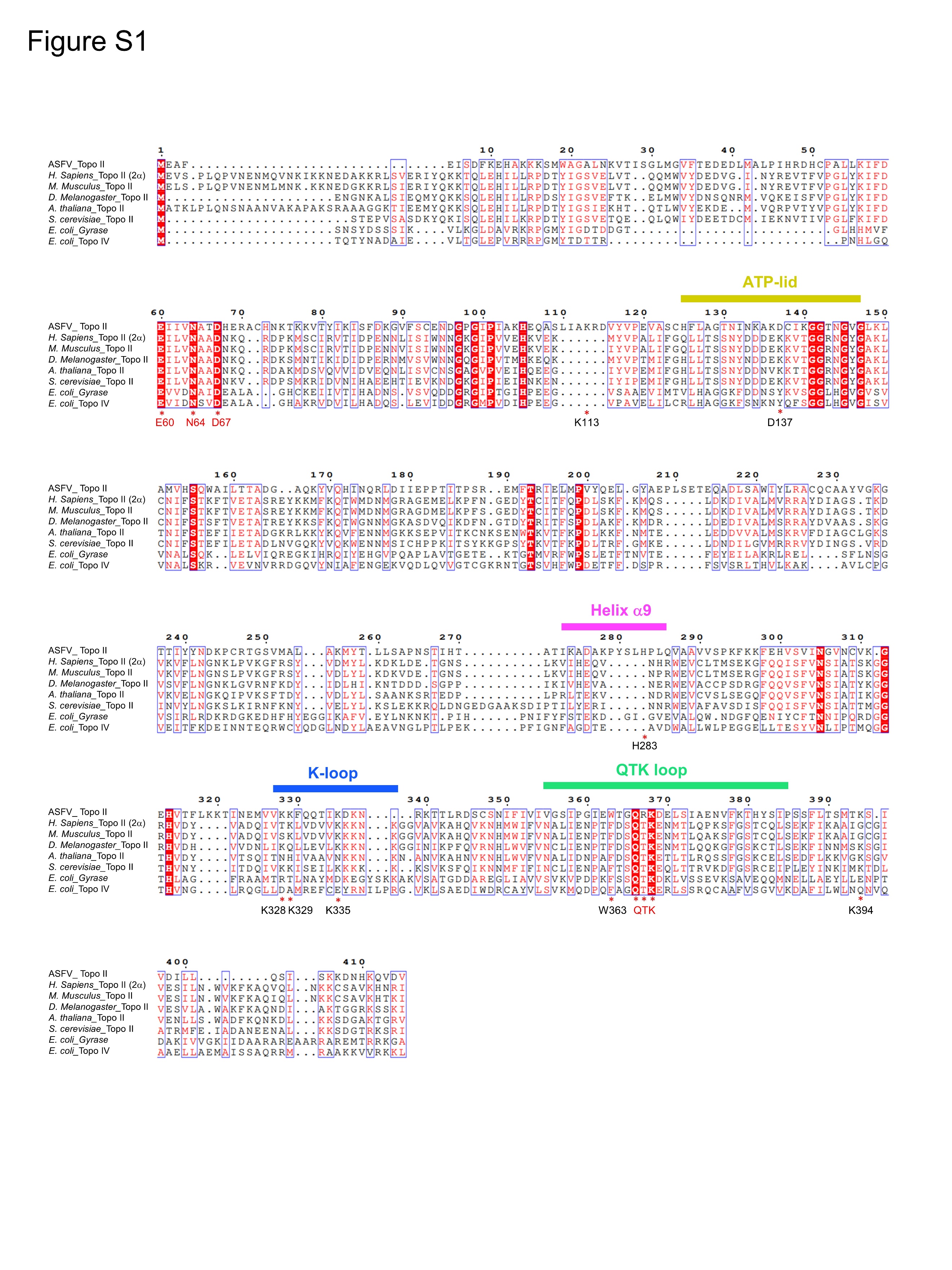


**Figure S1. Sequence alignment of the ATPase domains of ASFV topo II, eukaryotic and prokaryotic type IIA topoisomerases.** The special structural elements including ATP-lid, QTK loop, helix α9, and K-loop are indicated with colored straight lines. The highly conserved residues involved in Mg^2+^ and AMPPNP binding, and residues selected for mutation are indicated and colored in red and black, respectively.


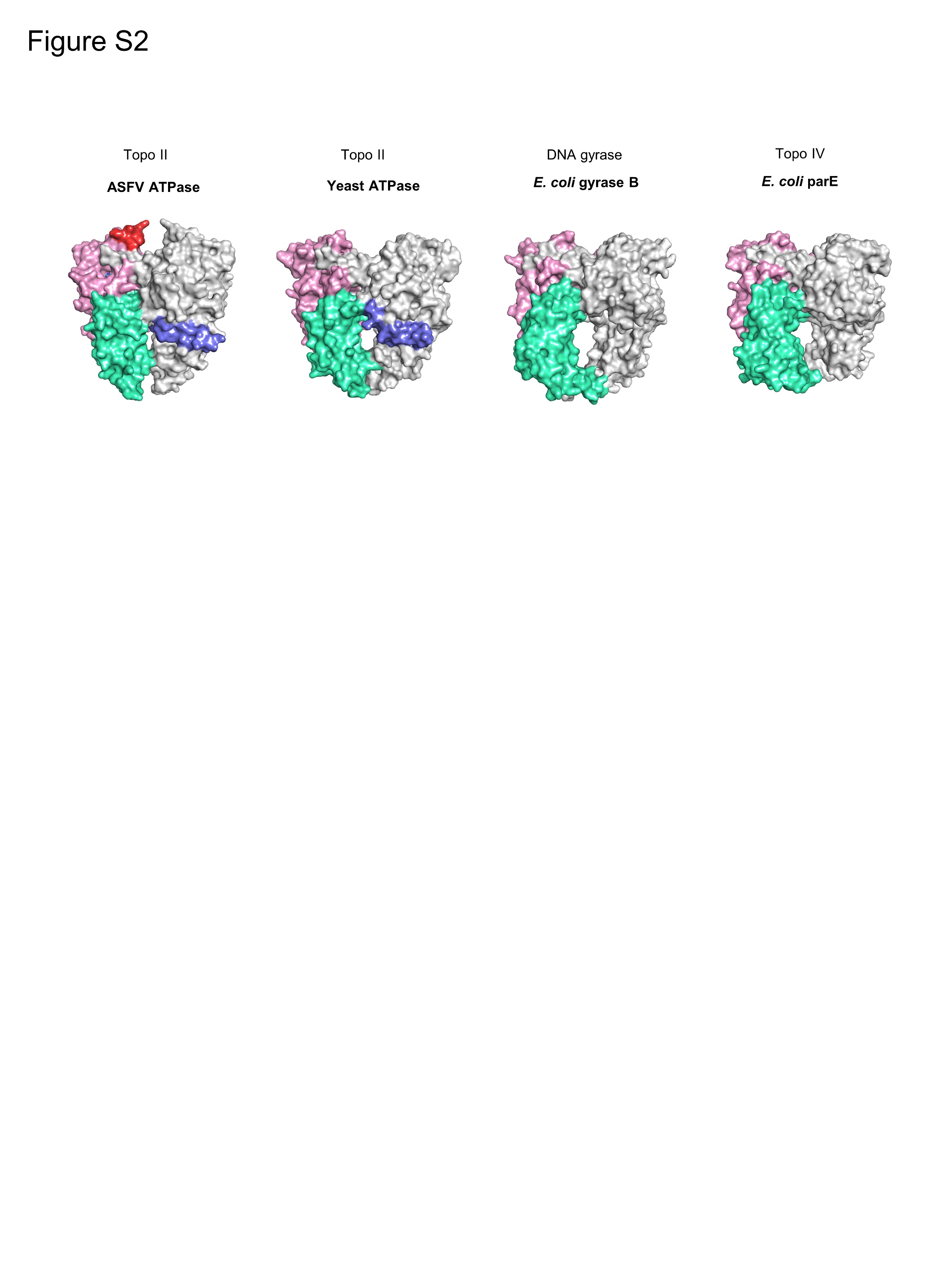


**Figure S2. Surface representation of ASFV topo II and other type II topoisomerase ATPase domains.** One protomer is colored as in Fig. 1A and the other is shaded gray. All the molecules are shown in the same orientation. The β-hairpin specific to ASFV and eukaryotic topo II is colored in slate.

**Figure S3.** **ICRF-187 has no inhibitory effect on the relaxation activity of ASFV topo II.** (A) Relaxation activity of WT and mutants. (B) Effect of ICRF-187 on the relaxation activity of WT ASFV topo II and mutants. ASFV topo II was incubated with pUC19 plasmids for 40 min at 30°C in the presence of increasing concentrations of ICRF-187. Reaction without enzyme was conducted as a negative control. The experiments were performed in triplicate, and the gel of one representative experiment is shown. Supercoiled (Sc) and relaxed (Rel) topoisomers are indicated. (C) Superposition analysis of ASFV topo II ATPase domain with yeast topo II ATPase-ICRF-187 complex. ICRF-187 bound in the dimer interface is shown as sticks and colored slate. One protomer is colored as Fig. 1A and the other protomer is colored gray. Residues involved in drug binding are indicated and shown as sticks. In ASFV topo II ATPase domain, residues M18 and W19 (T27 and Y28 in yeast topo II) with possible steric hindrance for ICRF-187 binding are highlighted with underlines.


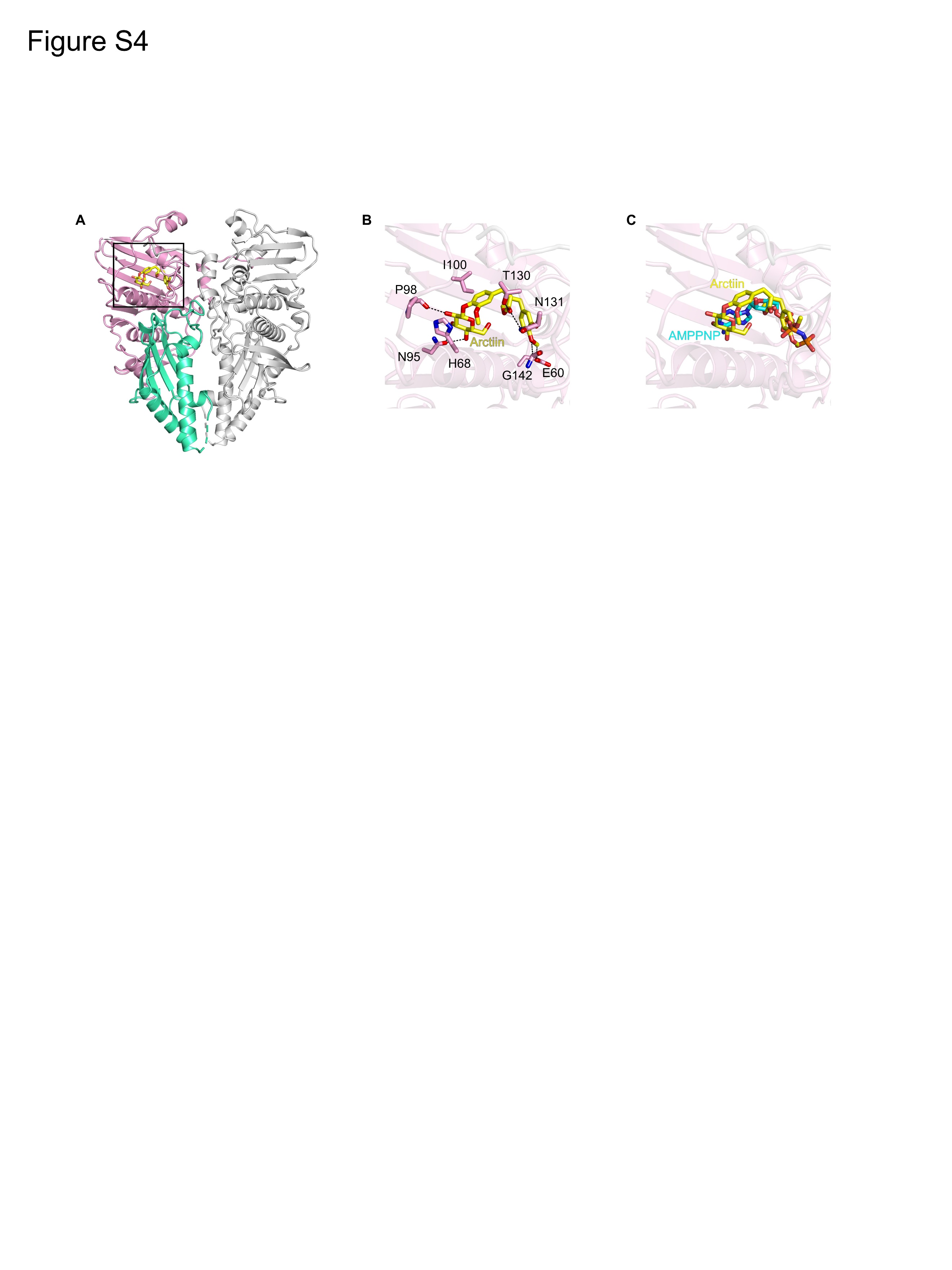


**Figure S4.** **Molecular docking of arctiin into the ATP binding domain of ASFV topo II ATPase domain.** (A) and (B) Molecular docking between arctiin and the ASFV topo II ATPase domain using AutoDock 4.2.6 program. One protomer is colored as in Fig. 1A and the other is shaded gray. Arctiin is shown in yellow stick. Key residues involved in arctiin binding are labeled and shown as sticks. (C) Comparison of the binding sites of AMPPNP and arctiin in the ASFV ATPase domain. The predicted binding site of arctiin is similar to that of AMPPNP.

**Table S1 Data collection and refinement statistics**

| PDB Code | ASFV topo II ATPase domain (8WWO) |
| --- | --- |
| **Data collection** |  |
| Space group | *P*3_1_ |
| Cell dimensions |  |
| a, b, c (Å) | 85.7, 85.67, 212.1 |
| α, β, γ (°) | 90.0, 90.0, 120.0 |
| Resolution (Å) | 50.00−2.20  (2.28−2.20) *^a^* |
| No. reflections | 84771 |
| *R*_merge_ | 0.100 (0.500) |
| *R*_meas_ | 0.120 (0.603) |
| CC*_1/2_* | 0.994 (0.731) |
| *I/σI* | 11.1 (2.2) |
| Completeness (%) | 96.5 (98.0) |
| Redundancy | 3.0 (2.9) |
| **Refinement** |  |
| Resolution (Å) | 2.20 |
| No. reflections | 84699 |
| *R*_work_/*R*_free_ (%) | 17.6/22.0 |
| No. atoms |  |
| Protein | 12261 |
| Ligand/ion /water | 124/4/1073 |
| *B*-factors (Å^2^) |  |
| Protein | 29.6 |
| Ligand/ion/water | 22.4/23.0/31.6 |
| R.m.s. deviations |  |
| Bond lengths, (Å) | 0.010 |
| Bond angles, (°) | 1.217 |
| Ramachandran statistics*^b^* | 93.2/6.2/0.6/0.0 |

*^a^*Values in parentheses are for highest-resolution shell.

*^b^*Values are in percentage and are for most favored, additionally allowed, generously allowed, and disallowed regions in Ramachandran plots, respectively.
